# Arthrodesis of the interphalangeal joints of the hand by two-dimensional intraosseous wiring

**DOI:** 10.1186/s12891-023-06972-z

**Published:** 2023-10-25

**Authors:** Tomoaki Suzuki, Daisuke Kawamura, Yuichiro Matsui, Norimasa Iwasaki

**Affiliations:** Department of Orthopaedic Surgery, Faculty of Medicine and Graduate School of Medicine, Hokkaido University, Nishi 5-chome, Kita 14-jou, Kitaku, Sapporoshi, Hokkaido 060-8648 Japan

**Keywords:** Two-dimensional intraosseous wiring (two-DIOW), Arthrodesis, Bone union, Surgical Complications

## Abstract

**Background:**

Numerous techniques for arthrodesis have been described to fix interphalangeal (IP) joints, and the fixation method should be considered on a case-by-case basis. This study aimed to investigate the availability of IP joint arthrodesis of the hand, using a two-dimensional intraosseous wiring (two-DIOW) method.

**Methods:**

A total of 43 joints (19 thumb IP joints, 9 proximal finger interphalangeal (PIP) joints and 15 distal interphalangeal (DIP) joints in 29 patients with a mean age of 66 years (range, 24–85 y) were retrospectively analyzed. All operations were performed with two-DIOW method. We evaluated the bone union rate, correction loss, presence of any surgical complications, and oral steroid use in cases of joint fixation using the two-DIOW method.

**Results:**

Of these 43 digits, 42 achieved bone union (97.7%). Non-union was seen in a thumb IP joint of mutilans rheumatoid arthritis. Mean correction loss of deviation was 1.0°, and flexion or extension angulation was 1.6° in the direction of extension. Surgical complications included mild nail deformity in 2 digits and wire irritation necessitating wire removal in 2 digits. Oral steroids were used for 18 of the 43 digits, including 2 digits complicated by nail deformities. There was no infection and skin necrosis in all digits with or without steroid use.

**Conclusions:**

The two-DIOW method appears to offer an effective method of IP joint fixation, but caution should be exercised in digits of severe joint destruction and in the treatment of wire knot.

**Supplementary Information:**

The online version contains supplementary material available at 10.1186/s12891-023-06972-z.

## Background

Arthrodesis of the interphalangeal (IP) joint is often necessary to treat pain, deformity, or instability associated with osteoarthritis (OA), rheumatoid arthritis (RA), or traumatic conditions. Numerous techniques for arthrodesis have been described, including single and crossed K-wire methods, intraosseous wiring [1], plates [2], staple [3], screws [4], and external fixation [5], up to a few years ago. These techniques provide stabilization of the joint, but offer limited compression of the fusion surfaces. Previous studies have reported rates of around 20% for major postoperative complications, including nonunion, malunion, osteomyelitis, and deep wound infections. More recently, fixation using various compression screws has become increasingly common for IP arthrodesis [6–9]. Nonunion is reportedly less frequent among patients treated with screw arthrodesis, compared to patients who underwent K-wire arthrodesis. On the other hand, compression screw techniques show some risks, such as difficulty with re-fixation due to bone defects at the time of nonunion, difficulty achieving fixation at free angles, prominent hardware, and cost. Two-dimensional intraosseous wiring (two-DIOW) represents a modification of intraosseous wiring first reported by Lister [10]. This modified procedure was originally reported in the Japanese literature for the fixation of phalangeal fractures to provide sufficient stability for early active motion of adjacent joints [11]. We hypothesized that this two-DIOW method would be applicable to arthrodesis of the IP joints of the hand. The purpose of this study was to investigate the clinical results of joint arthrodesis using the two-DIOW method and its application.

## Materials and methods

We used the two-DIOW method for thumb IP and finger proximal interphalangeal (PIP) and distal interphalangeal (DIP) joint arthrodesis between 2010 and 2016. A minimal follow-up period of 3 months was required for inclusion in the study. There were 43 digits in 29 patients, 4 men and 25 women, with a mean age of 66 years (range, 24–85 y). Arthrodesis was indicated for RA in 22 digits, OA in 17 digits, and posttraumatic arthritis in 4 digits (Table 1). Nineteen thumbs, 11 index fingers, 6 middle fingers, 5 ring fingers, and 2 little fingers were included. The joints were 19 thumb IP joints, 9 PIP joints and 15 DIP joints of the index-little finger. We obtained approval from our institutional review board and informed consent from the patients.

**Table 1:** preoperative diagnosis of patients

*Preoperative diagnosis*	*Patients(n)*	*Joints(n)*
Rheumatoid arthritis	14	22
Osteoarthritis	12	17
Posttraumatic arthritis	3	4
Total	29	43

Surgical technique.

We performed a dorsal curved or Y-shaped incision for IP and DIP joint and longitudinal incision for PIP joint. DIP joint was exposed by transection of the extensor tendon and collateral ligaments. PIP joint was exposed by longitudinal split of the extensor tendon and release of the collateral ligaments. The joint exposed in hyperflexion and the synovium, articular cartilage, and subchondral bone were resected using a rongeur to fit the desirable position at 0° to 30° flexion. We chose an angle of 0° to 25° at the thumb IP joint, 10° to 25° at the DIP joint, and 10° to 30°at the PIP joint.

**Fig. 1:**
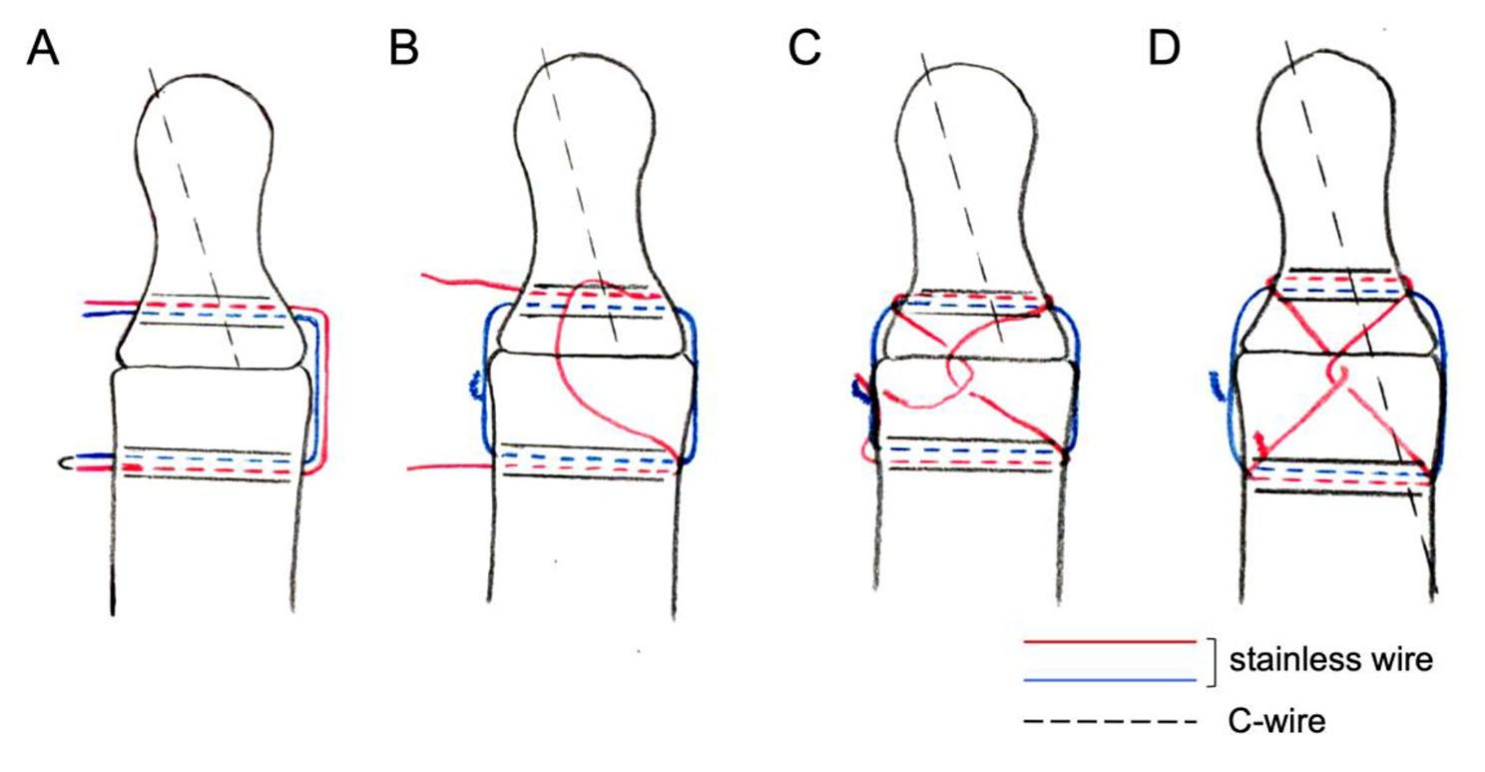
Illustration of two-DIOW method. **A** twofold stainless wire was passed through the same drill holes for cerclage wiring and then the looped end was removed. One or two C-wire should be pulled out retrogradely and distally from the resected articular surface. **B** One of the two wires was tensioned and twisted at a lateral side of the bones. **C** Another wire was placed at the dorsal aspect to make a cross with the same wire on the other side and then twisted. **D** Advance the C-wire from distal to proximal, penetrating the bone cortex

The two-DIOW method uses two stainless wires passed through the same bone holes for cerclage wiring. The bone holes are created with a C-wire (0.9-1.1 mm), a type of Kirschner wire (K-wire), in parallel at 3–5 mm from the resected articular surface. One or two C-wire should be pulled out retrogradely and distally from the resected articular surface. A twofold stainless wire (0.3-0.38 mm) was passed through the same bone holes and then the looped end was removed. One of the two wires was tensioned and twisted at a lateral side of the bones. Another wire was placed at the dorsal aspect to make a cross with the same wire on the other side and then twisted. Advance the C-wire from distal to proximal, penetrating the opposite bone cortex (Fig. 1). All skin incisions were closed with nylon sutures. The treated joint was splinted, and patients were encouraged to mobilize the adjacent joints immediately after surgery. Splint was continued for 4–6 weeks. The patients were allowed to use the hand in daily life, but were not allowed to use for heavy work until bone fusion was achieved.

We evaluated the bone union rate, correction loss, presence of any surgical complications, and associations between steroid use and complications. Radiographic union of the arthrodesis site was defined as bridging callus on at least 3 cortices from anteroposterior and lateral views, as interpreted by two orthopaedic surgeons. Bone union was evaluated every month up to 6 months postoperatively, and if bone union was not achieved at 6 months, confirmation was continued every 2 more months, until bone union was confirmed. In addition, we compared postoperative and post-bone union radiographs to measure the correction loss angle of radial or ulnar deviation in the anteroposterior view and flexion-extension angulation in the lateral view.

## Results

Bone union rate was 97.7%, with 42 of the 43 digits achieving bone union. Non-union was seen in only one digit: at the IP joint of a thumb with mutilans -type RA that required re-operation (Table 2). Three digits presented with erosive osteoarthritis of the DIP joints and required over 6 months to achieve final fusion. Mean correction loss of deviation was 1.0° (range, 0–4°), and flexion or extension angulation was 1.6° (range, 0–8°). No digits showed flexion dislocation exceeding 5°, but extension dislocation more than 5° was seen in 5 digits. Mild nail deformity, a longitudinal groove on the nail plate, was observed in 2 digits, both involving DIP joint with erosive osteoarthritis. Wire removal was required in 2 digits due to irritation by the intraosseous wire knot, and these were removed at 6 and 8 weeks postoperatively. In 2 cases of osteoarthritis, bony spurs on the adjacent digits caused irritation. Eighteen of the 43 digits were taking 2-5 mg of steroids orally for RA or other medical conditions, and 2 digits of them complicated by nail deformities. There was no infection in all digits with or without steroid use.

**Table 2:** Incidence of nonunion and delayed union following arthrodesis

		*Joints(n)*	*Nonunion(n)*
Thumb	IP	19	1
Index	PIP	4	0
	DIP	7	0
Middle	PIP	1	0
	DIP	5	0
Ring	PIP	2	0
	DIP	3	0
Little	PIP	2	0
	DIP	0	0

## Discussion

**Fig. 2:**
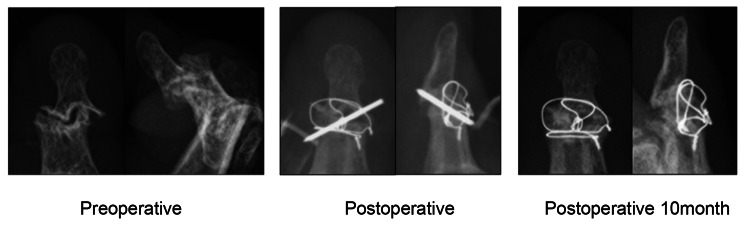
DIP joint fixation for mutilans RA. Two-DIOW method was performed for patients with severe DIP joint destruction by mutilans OA. Fixed at 3° radial deviation and 3° extension. 10 months postoperatively with no corrective loss and bone union

IP fixation is applied for the purpose of improving pain, correcting deformity, and addressing instability in cases where conservative treatment of digits affected by RA or OA proves ineffective. Close bone-to-bone contact and strong fixation are necessary to achieve bone union. Invasive fixation has been reported to offer a higher fusion rate than percutaneous fixation. Many techniques have been reported to date, varying the methods applied to the formation of bone and the materials used to provide fixation. We applied the two-DIOW method, which has been reported as an osteosynthesis method for phalanx fractures, to arthrodesis. The two-DIOW method is a rigid 2-dimensional fixation method that allows early exercise after bone fracture. This method shows features such as bone-to-bone compression, small bone defects, and no interference with flexor tendons, which are respectively considered superior to cross-pinning methods, compression screw methods, and interosseous wiring methods. In previous reports, the union rate of IP arthrodesis with these fixation methods has been described as 85–100% [6, 10, 12–15], and the results of 97.7% (42/43 digits) in our study were not markedly different. The one digit with nonunion comprised a thumb with mutilans-type RA, involved a distal phalangeal defect. Three of the DIP and IP cases required more than 6 months to achieve union. DIP cases are often accompanied by bone defects (Fig. 2), which may result in insufficient bone contact. In addition, tension band wiring can prove unstable due to the position of wire insertion [16]. The difficulties of wire procedures on the distal phalanx might thus contribute to inadequate fixation.

Mean loss of correction angle in the longitudinal direction was 1.0°, with no displacement exceeding 5°, and a fixed angle was generally maintained. On the other hand, mean loss of correction angle in the coronal direction was 1.6°, with 5 cases exceeding 5° in the direction of extension. We added all cases with C-wires, which were added as needed in the original method, because the two-DIOW method is slightly weak in the direction of extension. Removal of C-wires in the 5 cases with loss of correction angle exceeding 5° in the direction of extension was rather early, suggesting that a decrease in fixation force following early removal of the pin could have led to correction loss [12]. Because intraosseous wires can cause irritation, manipulating the inserted wire to as to avoid removal is important.

As postoperative complications in our study, nail plate deformity and irritation by the intraosseous wire knot were observed. In previous reports, complications have included bone spur pain with the cross-pinning method, infection, nail plate deformation, screw pain, and instability with the compression screw method, and skin necrosis and infections with the interosseous wiring method. Nail deformities were caused by nail matrix injury from wire fastening in cases with a bone defect in the phalanx due to erosive OA. As for irritation by the wire knot, this was attributable to contact with the bony spurts of adjacent digits in many OA cases. To prevent wire knot irritation, sufficient osteotomy is recommended, and use of a thinner and softer wire may suffice as possible.

Comparing cases with DIP and IP joint involvements to those involving PIP joints, the latter showed better results (Table 3). This finding is similar to the results of a previous study in which the nonunion rate was higher for DIP arthrodesis than for PIP arthrodesis [17] and another in which the tension band technique was useful for PIP arthrodesis [18]. In addition, when RA was compared to OA and post-traumatic arthritis, the bone union rate and the frequency of complications were almost the same (Table 4). Several previous reports comparing arthrodesis for OA and RA have found similar bone fusion rates and clinical outcomes [19, 20]. The comparable results in RA and OA in this study also suggest that two-DIOW may be a better indication for either condition.

**Table 3:** Comparison of DIP・IP joints and PIP joints

		*DIP & IP* *(n = 34)*	*PIP* *(n = 9)*
Bone union rate		97%	100%
Complication	Nail deformity	2	0
	Wire irritation	1	1

**Table 4:** Comparison of RA and others (OA and post-traumatic)

		*RA* *(n = 22)*	*Others* *(n = 21)*
Bone union rate		95%	100%
Complication	Nail deformity	1	1
	Wire irritation	0	2

In addition, it has been reported that K-wires are more cost-effective than commercial implants [21], and for the same reason, wires may be preferable for joint fixation from a healthcare system perspective.

Two key points for potential improvement were identified in those studies. First, pin manipulation is required to ensure removal is not needed. Second, to prevent wire knot pain, removal of sufficient bone marrow by osteotomy is warranted, and thinner and softer wires should be applied.

Our study has some limitations. First, its retrospective nature makes it difficult to make direct comparisons with other studies. Second, our radiographs were obtained at non-standardized intervals, thus making a determination of time to healing unreliable. Third, a minimum of 3 months may not be sufficient to identify late complications. Fourth, our patients had a broad array of diagnoses, which limits our ability to elucidate different subgroup characteristics.

## Conclusions

In this study, IP joint fixation using the two-DIOW method was shown to provide a good bone fusion rate without complications of skin necrosis or infection, regardless of steroid use. The two-DIOW method was suggested to be potentially useful in all IP joint fixations, although caution should be exercised for mutilans-type RA and erosive OA.

### Electronic supplementary material

Below is the link to the electronic supplementary material.

Supplementary Material 1

## Data Availability

The datasets used and/or analysed during the current study available from the corresponding author on reasonable request.
