# Correction: Arthrodesis of the interphalangeal joints of the hand by two-dimensional intraosseous wiring

**DOI:** 10.1186/s12891-024-07504-z

**Published:** 2024-05-16

**Authors:** Tomoaki Suzuki, Daisuke Kawamura, Yuichiro Matsui, Norimasa Iwasaki

**Affiliations:** Department of Orthopaedic Surgery, Faculty of Medicine, Graduate School of Medicine, Hokkaido University, Nishi 5-chome, Kita 14-jou, Kitaku, Sapporoshi, 060-8648 Hokkaido Japan

**Correction: BMC Musculoskelet Disord 24, 843 (2023)**

10.1186/s12891-023-06972-z

Following publication of the original article [1], the authors corrected a mistake in cited references (details of reference [11] is not correct).

Correct details of reference [11]:

Moriya K, Yoshizu T, Maki Y, Tsubokawa N, Narisawa H. New internal fixation technique of the digital skeleton in the hand; clinical results of two-dimensional intraosseous wiring. Orthopedic surgery [Jpn]. 2012;63:9–13.

Incorrect details of reference [11]:

Burton RI, Margles SW, Lunseth PA. Small-joint arthrodesis in the hand. J Hand Surg. 1986;11:678–82.

As a consequence to this amendment, the citation in the discussion part has been corrected from “[6, 10–15]” to “[6, 10, 12–15]”.

The original article [1] has been updated.
